# Hybrid antigens expressing surface loops of BauA from *Acinetobacter baumannii* are capable of inducing protection against infection

**DOI:** 10.3389/fimmu.2022.933445

**Published:** 2022-08-15

**Authors:** Somshukla Chaudhuri, Iraj Rasooli, Ramin Hatefi Oskouei, Mahdi Pishgahi, Abolfazl Jahangir, Vahid Farshchi Andisi, Anthony B. Schryvers

**Affiliations:** ^1^ Department of Microbiology, Immunology and Infectious Diseases, Cumming School of Medicine, University of Calgary, Calgary, AB, Canada; ^2^ Department of Biology, Shahed University, Tehran, Iran; ^3^ Applied Biotechnology Research Center, Baqiyatallah University of Medical Sciences, Tehran, Iran

**Keywords:** *Acinectobacter baumannii*, hybrid antigen design, acinetotbactin, BauA siderophore receptor, TonB dependent transport system, vaccines

## Abstract

*Acinetobacter baumannii* is a human bacterial pathogen of increasing concern in clinical settings due to the emergence of antibiotic resistant strains and the lack of effective therapeutics. Researchers have been exploring new treatment options such as novel drug candidates and vaccines to prevent severe infections and mortality. Bacterial surface antigens that are essential to *A. baumannii* for acquiring micronutrients (e.g. iron, zinc) from nutrient restricted environments are being considered as targets for vaccines or immunotherapy due to their crucial role for growth and pathogenesis in the human host. BauA, the outer membrane receptor for the siderophore acinetobactin was targeted for vaccine development in this study. Due to challenges in the commercial production of membrane proteins for vaccines, a novel hybrid antigen method developed by our group was used. Exposed loops of BauA were selected and displayed on a foreign scaffold to generate novel hybrid antigens designed to elicit an immune response against the native BauA protein. The potential epitopes were incorporated into a scaffold derived from the C-lobe of *Neisseria meningitidis* transferrin binding protein B (TbpB), named the loopless C-lobe (LCL). Hybrid proteins displaying three selected loops (5, 7 and 8) individually or in combination were designed and produced and evaluated in an *A. baumannii *murine sepsis model as vaccine antigens. Immunization with the recombinant BauA protein protected 100% of the mice while immunization with hybrid antigens displaying individual loops achieved between 50 and 100% protection. The LCL scaffold did not induce a protective immune response, enabling us to attribute the observed protection elicited by the hybrid antigens to the displayed loops. Notably, the mice immunized with the hybrid antigen displaying loop 7 were completely protected from infection. Taken together, these results suggest that our hybrid antigen approach is a viable method for generating novel vaccine antigens that target membrane surface proteins necessary for bacterial growth and pathogenesis and the loop 7 hybrid antigen can be a foundation for approaches to combat *A. baumannii* infections.

## Introduction

The Gram-negative coccobacillus *Acinetobacter baumannii* is an opportunistic human pathogen associated with numerous nosocomial conditions such as burn and wound infections, ventilator-associated pneumonia, and sepsis in critically ill patients (1, 2). Clinical isolates of *A. baumannii* can resist treatment by currently available antibiotics and can tolerate environmental insults including desiccation and oxidative stress (3, 4). The increase in resistant disease isolates has made it one of the ESKAPE pathogens recognized by the Infectious Diseases Society of America (IDSA) (5) and one of the priority antimicrobial resistant pathogens that warrant investigation of new therapeutic approaches by the WHO (6).

Several virulence factors have been identified in the *Acinetobacter* species including iron-uptake pathways (4). Iron is a micronutrient essential for the vertebrate host and almost all bacterial microbes as it is critical for various physiological functions including, but not limited to, serving as a cofactor in various enzymatic reactions, energy metabolism, oxygen transport, and gene regulation (7). Within the vertebrate host, iron is chelated by host iron binding proteins, therefore, the level of free extracellular iron is incredibly low (8). This iron-sequestration strategy maintains the concentration of free iron at levels that are insufficient in supporting bacterial survival and growth thus suppressing bacterial infections, a process known as nutritional immunity (9, 10).

To survive under iron-limited conditions *in vivo*, bacteria residing in mammalian host environments must possess strategies for acquiring iron from the host to meet their own iron requirements (11). One of the primary methods for bacterial iron acquisition is through siderophore chelation; secretion of high affinity iron chelators that sequester iron and the resulting iron-siderophore complex is captured and utilized by the bacteria (12). In Gram-negative bacteria like *A. baumannii* the iron-siderophore complex is bound by a specific siderophore receptor that couples to the TonB system to translocate the complex (and thus iron) into the periplasm where it is bound by a periplasmic binding protein and then transported across the inner membrane by an ABC transporter (13). Iron uptake pathways expressed by *A. baumannii* have attracted considerable attention for the development of novel therapeutics. *A*. *baumannii* can possess 3 different loci encoding up to ten distinct siderophores: acinetobactin and preacinetobactin (known together as acinetobactin), baumannoferrins A and B, and fimsbactins A-F (14). However, fimsbactins are only present in a small subset of strains (15). While all three siderophore associated loci are known to be upregulated during iron restriction *in vitro* and *in vivo*, it is in fact the inactivation of the acinetobactin biosynthesis pathway that attenuates survival of *A*. *baumannii* in a murine model (14). Additionally, the acinetobactin locus is highly conserved amongst *A. baumannii* clinical isolates (14, 16) further suggesting that acinetobactin is critical to supporting *A. baumannii* survival and virulence. Thus, designing novel therapeutics that target this essential siderophore system can represent an alternative avenue for neutralizing this pathogen.

The receptor for acinetobactin uptake is outer-membrane transporter protein baumannii acinetobactin utilization A, BauA for which the structure has recently been solved (17). BauA has the typical outer-membrane TonB dependent transporter (TBDT) architecture with a ß-barrel formed by 22 anti-parallel beta-strands and a plug domain folded inside of the barrel. Complementing studies demonstrating the importance of acinetobactin synthesis in virulence in mouse infection models (14, 16) and the observation that deletion of BauA also leads to reduced virulence (16) argue for it being logical target for vaccines. The demonstration that intact BauA was protective in a mouse infection model provides support for pursuing vaccines targeting this antigen (18).

Although TonB-dependent receptor proteins involved in nutrient acquisition may be ideal targets for vaccine development, there are significant challenges for commercial production as they are normally produced in the outer membrane in much lower yields. Due to the large hydrophobic surfaces embedded in the membrane, detergents are required for extraction from the membrane or for solubilizing the protein generated by other methods (19). In addition, the important surface epitopes that would be the target of effective antibodies are formed by the extracellular loop regions of the beta-barrel, thus it is desirable to focus the immune response on those regions.

A hybrid antigen approach was developed to target the surface loops of the TBDT transferrin binding protein A (TbpA) from *Neisseria meningitidis* by displaying them on the ‘scaffold’ derived from the surface lipoprotein (SLP) transferrin binding protein B (TbpB) which contains similar anti-parallel beta strand structures that are soluble in aqueous solution (19). The rationale for this approach was that splicing the TBDT surface loops onto the SLP scaffold would provide similar conformational constraints on the loops, thus increasing the potential to generate antibodies that bind to the native conformation in the TBDT. Mice immunized with these antigens elicited antibodies that recognized the native protein expressed by whole cells and exhibited complement mediated bactericidal activity. Separate immunization and challenge experiments demonstrated protection against *N. meningitidis* in a mouse sepsis model and against colonization by *N. gonorrhoeae.* However, the scaffold derived from the C-lobe of TbpB induced antibodies against *N. meningitidis* thus strategies such as using strains defective in expressing TbpB were required to evaluate loop specific responses.

To evaluate whether this approach was generally suitable for TBDTs, the foreign ‘loopless C-lobe’ (LCL) scaffold derived from *N. meningitidis* TbpB was used to display the surface loops from an *A. baumannii* TBDT inadvertently mis-identified as ZnuD (20). The authentic *znuD* gene (genomic locus A1S_2892, strain ATCC 17978) was identified in an elegant study (21) demonstrating its ability to overcome calprotectin-mediated zinc sequestration. However, the closest homologue in a BLAST search of the available genomic sequence for strain ATCC 19606 at that time was locus eex02556.1 and was used for computational modelling and the design of hybrid antigens. Unfortunately, of the five available scaffold level genomes that are now available for strain ATCC 19606, only one is positive in a BLAST search with the authentic *znuD* (*znuD1*) gene, highlighting the challenges of using genome assemblies that are not closed with sequence data. Nevertheless, mice immunized with the hybrid ‘ZnuD’ antigens were protected against lethal challenge by *A. baumannii* while the foreign scaffold did not provide any protection, demonstrating the utility of the hybrid antigen approach for evaluating loop specific protection. The gene encoding this TBDT that can be identified in genomic sequences with the primer sequences used for hybrid gene construction (20) is distinct from the authentic ZnuD that can be identified by primers used for qPCR in the original study (21) but the specific function of this protein has not been determined.

In the current study we opted to use the foreign LCL scaffold for display of epitopes from *A. baumannii* BauA as the scaffold would not be expected to induce any antibodies against *A. baumannii* that could complicate the analysis of results. Our hybrid antigens were originally designed using a computationally derived model for BauA but the subsequent availability of structures derived from protein crystallography (17) confirmed the design and assisted in the interpretation of the results. The hybrid antigens were used in immunization and challenge experiments to determine the nature and effectiveness of the immune response. Our results demonstrate that immunization with hybrid antigen displaying a single BauA loop (loop 7) was able to protect mice during a lethal challenge by *A. baumannii* as effectively as immunization with recombinant BauA. This suggests that our hybrid antigen approach may be a viable method for generating novel vaccine antigens that target membrane surface proteins necessary for bacterial growth and pathogenesis.

## Material and methods

### Computational modelling, loop selection and design of hybrid antigens

**Figure 1:**
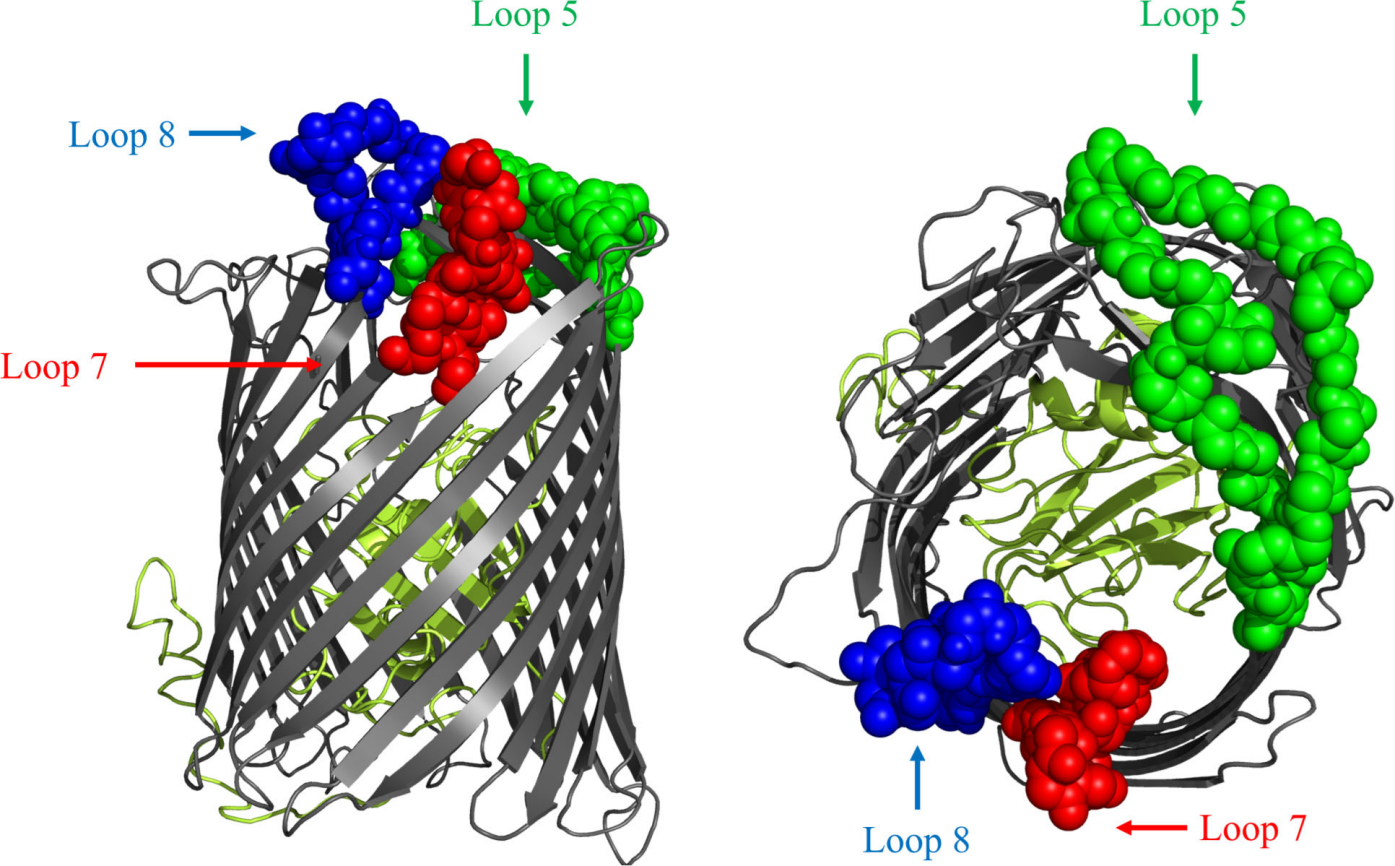
Cartoon diagram of a computational structural model of BauA from *A. baumannii* AAT52186.1 generated with I-Tasser. The plug region is coloured in olive and the beta-barrel is coloured dark gray. The regions of the three loops selected for display on the LCLv3 scaffold are labeled as spheres and are coloured representing the three selected loops. Left image is side view. Right image is top-down view. Loop 5 - green, Loop 7 - red, and Loop 8 – blue.

The design of hybrid antigens was completed prior to the availability of the crystal structures of BauA (17). After removing the predicted signal peptide region with the SignalP program (22), computational approaches were used to develop structural models for BauA with sequence AAT52186.1 using I-Tasser (23). The resulting model was visualized in PyMOL to determine the ‘anchoring position’ of the extracellular loops of this TonB-dependent outer membrane surface antigen (**Figure 1**) (24). Loops 5,7, and 8 were selected for the design of hybrid antigens using a combination of bioinformatic epitope analysis (25) and examining the structural model to identify well exposed extracellular loops.

**Figure 2:**
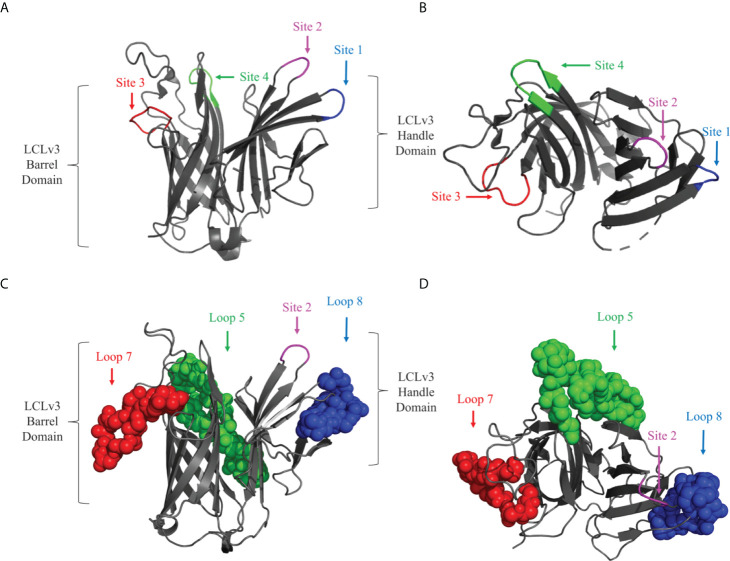
Cartoon diagram of the LCLv3 scaffold derived from the crystal structure of the *N. meningitidis* loopless C-lobe (PDB 5KKX). **(A)** The side view and **(B)** top view of the LCLv3 scaffold alone with the four sites labelled. Site 4 - green, Site 2 – Magenta, Site 3 – red, and Site 1 – blue. **(C)** The side view and **(D)** top view of LCLv3 with BauA loops depicted as spheres. Loop 5/site 4 - green, Loop 7/site 3 - red, and Loop 8/site 1– blue. The handle and the barrel domain of the LCLv3 are also labelled.

A previously described foreign scaffold (19) was used to display selected BauA loops. Briefly, a derivative of the TbpB C-lobe from *N. meningitidis* strain M982 was employed for displaying the selected BauA loops. Originally, the ‘loopless C-lobe’ (LCL) (19) was generated when the larger loop regions in the C-lobe were not resolved in the crystal structure (26) and thus were replaced with equivalent short linking regions identified in the structure of an *Actinobacillus pleuropneumoniae* TbpB (27). In this manuscript, a modified LCL (LCLv3) that contains regions from two *N. meningitidis* TbpBs (28) was used and foreign loops were inserted into sites 1-4, created by replacing TbpB loops 21, 23, 27, and 31 respectively from the scaffold (**Figures 2A, B**). BauA extracellular loop 8 ^554-576^, loop 7 ^507-526^ and loop 5 ^396-435^ (**Figure 1**) were selected for insertion into sites 1, 3 and 4 of LCLv3 respectively (**Figures 2C, D**). The loop7 hybrid antigen has also been used in a recently published study (29).

**Table 1:** Primers sequences used in this study.

Amplicon name	Primer sequence	Size (bp)
**BauA**	**F:**ACTG **GGATCC** ATGAATGATCAAAGAATTAAT **R:** GTTT **CTCGAG** TTAAAAGTCATATGATACAGATAGCATA	2178
**Loop 5**	**Loop5F:** ggtttgcgtatGATTATGGTTATAGAATTATCCC **Loop5R:** gacgaccgttgcAAATAGAAACGGCGGTGT	134
**Loop 7**	**Loop7F:** agaaaattaccggtaaactgacgAAGGGTGATCAGGCACC **Loop7R:** gccttcaatcgtaaaggtAGGAGGAAATATTTCCCC	95
**Loop 8**	**Loop8F:** ttacccgcaaatttgaacacacgTATTTAGATCCTTCAAAGC **Loop8R:** aacagcagacttcgacttcataggtCGAAACAAAGGTAGGTAAG	96
**Vector specific forward**	**577:** GTCGTCAGACTGTCGATGAAGCC	–
**Vector specific reverse**	**2831:** ATGAGGCTGCGGTTGCGTTCCTG	–
**Position 1**	**Pos1R:** AGGATCTAAATAcgtgtgttcaaatttgcg **Pos1F:** CCTTTGTTTCGacctatgaagtcgaagtctgctg	284 701
**Position 3**	**Pos3R:** GATCACCCTTcgtcagtttaccggtaattttcttg **Pos3F:** ATTTCCTCCTacctttacgattgaaggcatgatc	573 400
**Position 4**	**Pos4R:** ATAACCATAATCatacgcaaaccaaccgcccag **Pos4F:** CCGTTTCTATTTgcaacggtcgtcttcggtgc	782 183

Small letters on primer sequences correspond to LCLv3 sequences and capital letters correspond to loop positions. The bold and underlined letters correspond to restriction cut sites.

Primers (**Table 1**) were designed to the extracellular loop regions from BauA with the 5’ regions corresponding to the selected sites on scaffold. The loops were introduced into the three sites of the LCLv3 by SOE (splicing by overlap extension) PCR (30) so that they would encode hybrid antigens containing the individual loop regions (LCLv3-loop 5, LCLv3-loop 7, LCLv3-loop 8) or combinations of two or three loops together (LCLv3-Loops 85, LCLv3-Loops75, and LCLv3-Loops 875).

### Bacterial strains and plasmids

*A. baumannii* strain ATCC 19606 was used for sepsis experiments and as a source of genomic DNA for *bauA* gene sequencing. *Escherichia coli* strain Top10 was used for cloning protocols whereas *E. coli* strain ER2566, a BL21 derivative, was used for protein production. The bacteria were grown in Luria Bertani (LB) broth or on LB agar medium at 37°C supplemented with ampicillin (100 µg/ml) if the strains were carrying plasmids. Custom protein expression plasmid e5818 was used for protein production. Plasmid e5818 contains the T7 promoter, an N-terminal polyhistidine tag, a maltose binding protein (MBP), and a Tobacco etch virus (TEV) protease cleavage site followed by *Bam*HI and *Xba*I/*Hind*III cloning sites with a kanamycin resistance locus in between the *Bam*HI and *Xba*I sites.

### Production of BauA and the hybrid antigens

The *bauA* gene was amplified from the genomic DNA of *A. baumannii* ATCC 19606 and sequenced. After using Signal P (22) to identify the mature coding sequence of the *bauA* gene, the primers listed in **Table 1** were used to amplify a PCR product that was digested with BamHI and XhoI and cloned into plasmid pET28c. The gene fragment encoding LCLv3 (20) was digested with BamHI and XbaI and cloned into custom plasmid e5818. To insert the gene fragments encoding the *bauA* loops 5, 7 and 8 into the selected sites on LCLv3 (**Figure 2**), SOE -PCR (30) was used with primers listed in **Table 1**. The resulting gene fragments were digested with BamHI and XbaI and cloned into the custom expression vector e5818 and transformed into chemically competent *E. coli* Top10 and confirmed by sequencing.

The vectors with sequence confirmed LCLv3 and BauA-LCLv3 hybrid gene fragments were transformed into competent *E. coli* ER2566 cells. The soluble and insoluble proteins were produced as previously described (24). Soluble proteins were expressed by growth in autoinduction media at 37°C overnight in a shaking incubator. Cells were harvested the following day by centrifugation (10000×g/20 mins/4°C), resuspended in TE lysis buffer (Tris-HCL 10 mM, EDTA 1 mM), and lysed by sonication on ice for 20 min (with 70% resonance power at 30s interval). The crude lysate was separated from the cell debris by centrifugation (10000×g/20 mins/4°C) and then subjected to Ni-NTA chromatography and eluted off the column with an elution buffer containing 300 mM imidazole. Fractions were analyzed using sodium dodecyl sulfate-polyacrylamide gel electrophoresis (SDS-PAGE) to determine the purity of the sample. Eluted fractions were pooled, dialyzed against phosphate-buffered saline (PBS), and concentrated. Protein samples were filter sterilized (0.22 µm filter), quantified, and stored in aliquots at -20°C until use.

Proteins that were not readily detectable by SDS-PAGE analysis with the protocols described above were deemed insoluble, thus a procedure for isolation of protein from inclusion bodies was implemented (20, 24). ER2566 cells harboring the expression vectors were grown and pelleted as described above. The pellet was resuspended in buffer B (NaH_2_PO_4_ 100 mM, NaH_2_PO_4_ 10 mM, Urea 8 M, pH= 8.0) and incubated on ice for 15 minutes. The solubilized inclusion body fraction was separated from the cell debris by centrifugation (10000×g for 20 min at 4°C) was subjected to a NiNTA agarose column to capture the solubilized proteins. The column was exposed to decreasing concentrations of urea starting from a 6 M urea buffer going down to a 2 M urea buffer. The protein was eluted from the column with a 2 M urea and 300 mM imidazole containing buffer, dialyzed against PBS and subsequently analyzed by SDS-PAGE for purity. **Supplementary Table 1** summarizes the solubility characteristics of each hybrid antigen.

### Animal study and ethical clearance

Healthy male BALB/c mice aged 6-8 weeks and weighing 20-25 g were procured from the Pasteur Institute, Tehran, Iran. The animals were housed in a standard hygienic and well-ventilated environment with water and animal feed in the animal care facility at Shahed University. The research was conducted in accordance with the principles of the National Institutes of Health guide for the care and use of Laboratory animals (NIH Publication No. 8023, revised 1978) and with animal care guidelines confirmed by Animal Care and Ethical Committee of Shahed University (ethics certificate 1399.073 issued by Shahed University).

### Mouse immunizations

The experimental animals were divided into nine groups based on the immunization formulation with each group comprising of 6 mice. The nine immunization formulations administered to the mice were the adjuvant, LCLv3 scaffold alone, recombinant BauA, hybrid antigen displaying Loop 8 (L8), hybrid antigen displaying Loop 7 (L7), hybrid antigen displaying Loop 5 (L5), and hybrid antigens displaying different loop combinations (L75, L85, L875).

Mice were immunized subcutaneously (SC) with a prime and two boosters where each dose was given 14 days apart on Day 0, Day 14, and Day 28 of the experiment. Freund’s complete adjuvant (Sigma) was used for the prime followed by Freund’s incomplete adjuvant for the booster dosages. For each group, 100 µl of the protein-adjuvant mixture at 1:1 (v/v) ratio containing 20 µg protein was administered to the mice. The serum samples were collected one week after each dose for assessing systemic antibody titres.

### IgG antibody titre measurement by ELISA

Antisera collected from blood samples harvested at Day 7 (D7), Day 21 (D21), and Day 35 (D35), were used to quantify elicited IgG antibody titres against each immunizing antigen by enzyme-linked immunosorbent assay (ELISA). In brief, 96 well flat-bottom plates were coated with 5 µg/well of the immunizing antigen in carbonate-bicarbonate buffer (pH 9.6) at 4°C overnight for protein capture and immobilization. The following day, the plates were washed three times with 250 µL of PBS containing 0.05% Tween 20 (PBST). All wash steps mentioned henceforth were performed 3 times with 250 µL of PBST. Wells were blocked with 250 µl of 5% skim milk in PBST (w/v) for 45 minutes at 37°C and washed. 100 µL of antisera diluted in 2.5% skim milk in PBST was added in duplicate in 2-fold dilutions going from 1 in 250 to 1 in 512,000 and wells were incubated for 2 hours at 37°C and washed. 100 µL of goat anti-mouse IgG secondary antibody [conjugated to horseradish peroxidase (HRP)] (Sigma Aldrich, USA) diluted 1 in 15,000 in 2.5% skim milk in PBST was added to each well and incubated for 1 hour at 37°C and washed. Plates were developed using 50 μL of 3,3,5,5′-tetramethylbenzidine (TMB) reagent (Sigma Aldrich, USA) for 20 minutes in the dark. Development was quenched with 25 μL of 3 M H_2_SO_4_ and OD_450_ readings taken using a 96 well plate reader and plotted.

### Bacterial challenge with *A. baumannii* of immunized mice

Preparation of bacterial inocula of *A. baumannii* ATCC 19606 and performance of the challenge was performed essentially as described previously (20). Groups of 8 mice were infected with increasing bacterial dosages ranging from 10^4^ to 10^7^ CFU, supplemented with porcine mucin to determine the lethal dose (LD) of *A. baumannii* ATCC 19606. Three weeks after the last immunization, mice were challenged with an LD of 1.8×10^6^ CFU of *A. baumannii* ATCC 19606 intraperitoneally. Challenged mice were monitored for 168 hours for clinical symptoms and were euthanized when severely ill or at experimental endpoint.

### Passive immunization and bacterial challenge with *A. baumannii*

Antisera collected from the immunized groups of mice prior to challenge was pooled and heat-inactivated at 65°C for 30 minutes to neutralize endogenous complement activity. Three hours prior to an intraperitoneal challenge with the LD of *A. baumannii* ATCC 19606, groups of 6 naïve mice, aged 6 to 8 weeks old, were intravenously injected with 100 µL of the heat inactivated sera. Mice were monitored for 168 hours post challenge and euthanized when severely ill or at experimental endpoint.

### Evaluation of bacterial burden in mice organs

Groups of 6 mice were immunized with the different antigen formulations and challenged intraperitoneally with the lethal dose (1.8×10^6^ CFU) of *A. baumannii* ATCC 19606. 24 hours after challenge, all of the mice were euthanized and the lung, liver, and spleen were removed aseptically to determine the bacterial load. Each tissue was weighed and homogenized in 2 mL PBS, which was plated onto LB agar in serial dilutions and incubated at 37°C for 18-24 hours. The following day the number of CFUs were determined, and the results were expressed as log_10_ CFU.

### Statistical analyses

All data were analyzed using GraphPad Prism™ V8 or V9 (GraphPad Software, San Diego, California, USA). The comparative survival analysis was performed using a non-parametric log rank test. For bacterial burden on mice organs, statistically significant differences were determined by One-way analysis of variance (ANOVA) test followed by Holm-Sidak’s multiple comparison for normal data distribution. Statistically significant differences were determined by non-parametric Kruskal–Wallis test followed by Dunn’s multiple comparison where the data distribution was not normal. The mean immunoglobulin titres from ELISAs were analyzed for significance using two-way ANOVA followed by Tukey’s *Post Hoc* Analysis. Serological results are reported as means ± SD (standard deviation), and p-values < 0.05 were considered significant.

## Results

### Hybrid antigen design and production

To select surface exposed loops of BauA for displaying on the scaffold, a structural model of BauA using computational approaches was first generated with I-Tasser (**Figure 1**). To potentially select the best loops for display, we used a combination of considering prior bioinformatic B-cell epitope analysis (25) and evaluating the potential accessibility of loops in the structural model (**Figure 1**). Three loops were selected and displayed either individually or in combination to generate hybrid antigens for testing. The selected extracellular loop 5^396-435^, numbered from the mature N- terminus, (DYGYRIIPGFSDPVITNIYDPNPNWGPKPEFTPPFLF) and loop 7^507-526^ (KGDQAPATASNPGEIFPP) were transplanted onto sites 4 and 3 on the barrel domain of the LCLv3 scaffold (**Figures 2C, D**). Selected loop 8^554-576^ (YLDPSKLVNNLPTFVS) was transplanted onto site 1 on the handle domain of the LCLv3 scaffold (**Figure 2**). LCLv3 is a foreign scaffold derived from TbpB C-lobes from *N. meningitidis* (20, 24), which does not induce protective antibodies against *A. baumannii* (20) thus any ability to protect against infection can be attributed to the displayed epitopes. A total of six hybrid antigens were designed (**Supplementary Table 1**): 3 hybrids expressing single loops (L8, L7, and L5), two hybrids expressing two loops (L85 and L75), and one hybrid expressing all three loops (L875).

The hybrid antigen gene fragments were cloned into a custom vector encoding a T7 promoter for protein expression by an inducible T7 RNA polymerase. The vector also encodes an N-terminal polyhistidine tag, and TEV protease cleavage site to enable protein capture by a Ni-NTA column, aid in protein folding, and provide the option for removing the fusion construct through proteolytic activity, respectively. The T7 promoter driven expression system leads to high levels of protein expression in the cytoplasm but may result in the formation of inclusion bodies. The recombinant BauA antigen, hybrid proteins, and the LCLv3 scaffold were all expressed with the T7 promoter system and if the proteins were not soluble in high yields, a protocol for solubilizing the proteins from inclusion bodies was implemented. Only the vectors containing the genes encoding the LCLv3 scaffold or hybrid proteins displaying loops 8 and 7 provided sufficient soluble protein purified from the supernatant of lysed cells for subsequent experiments (**Supplementary Figures 1–3**, **Supplementary Table 1**) even with the N-terminal MBP present. For the remaining genes encoding hybrid antigens or the recombinant BauA protein, the inclusion bodies in the cell pellet were solubilized in buffers containing 8 M urea followed by capture with a Ni-NTA column (**Supplementary Figures 1–3**, **Supplementary Table 1**). The captured polypeptides were refolded by using decreasing concentrations of urea and then dialyzed against PBS.

### The protective effect of the immune response against the selected epitopes

Serum was sampled at day 7, day 21, and day 35, seven days after each immunization, and the IgG titres evaluated in ELISA assays with the immunizing antigen. All the antigens elicited reasonably high antibody titres after the third immunization (**Supplementary Figure 4**). Although it was not possible to determine the titre of IgG directed against individual loops, the LCLv3 or the N-terminal 43 kDa present in the immunizing antigens in this assay, prior studies had demonstrated that the response against LCL- had no protective effect against *A. baumannii* infection, thus would not interfere with interpretation of the challenge experiment (20).

**Figure 3:**
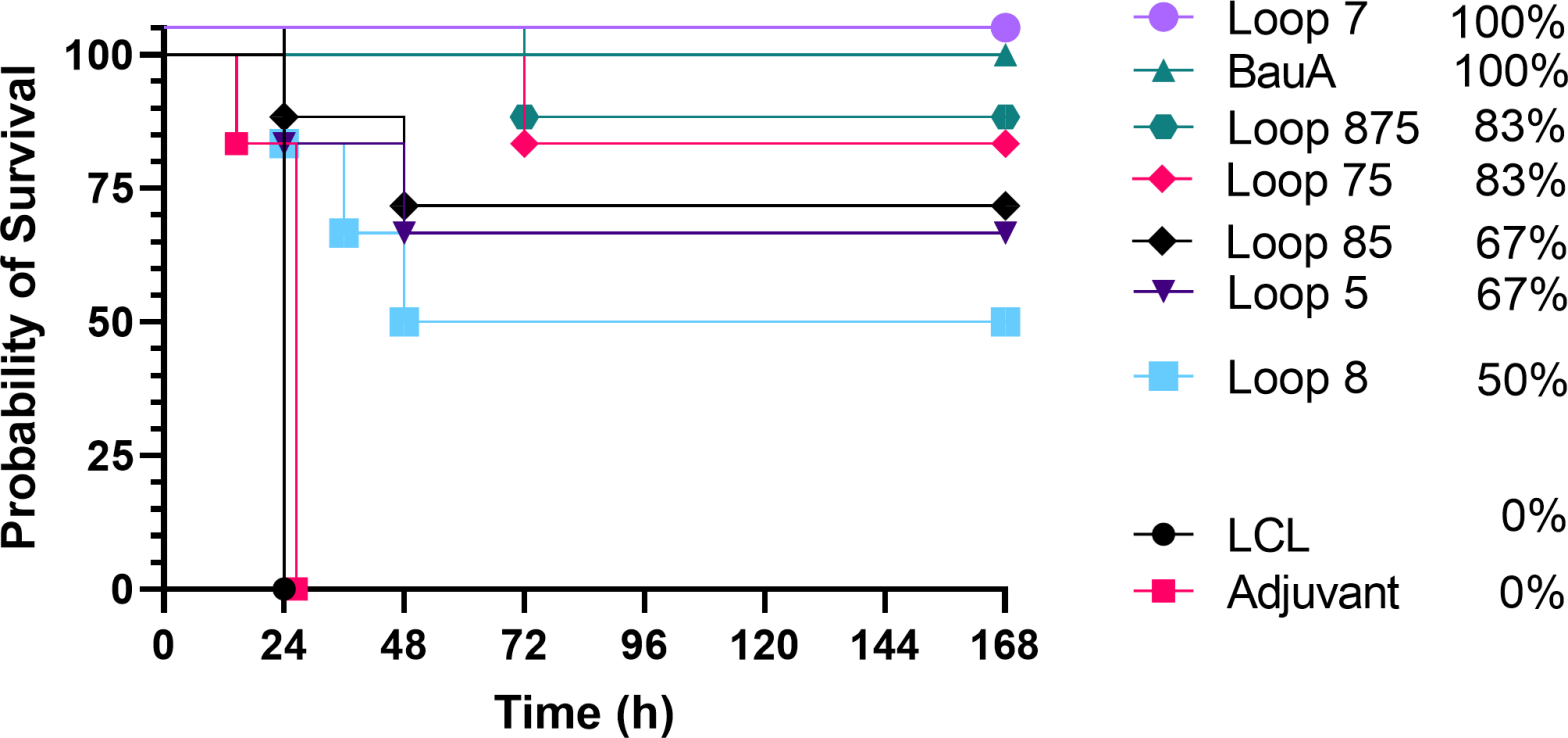
Survival curves of mice immunized with different antigens and challenged with a lethal dose of *A. baumannii* ATCC 19606. Mice were challenged with 1.8×10^6^ CFU and monitored for 168 hours (7 days) post challenge for symptoms. Survival curves were analyzed using a non-parametric log rank test and were significant (p<0.0001).

**Figure 4:**
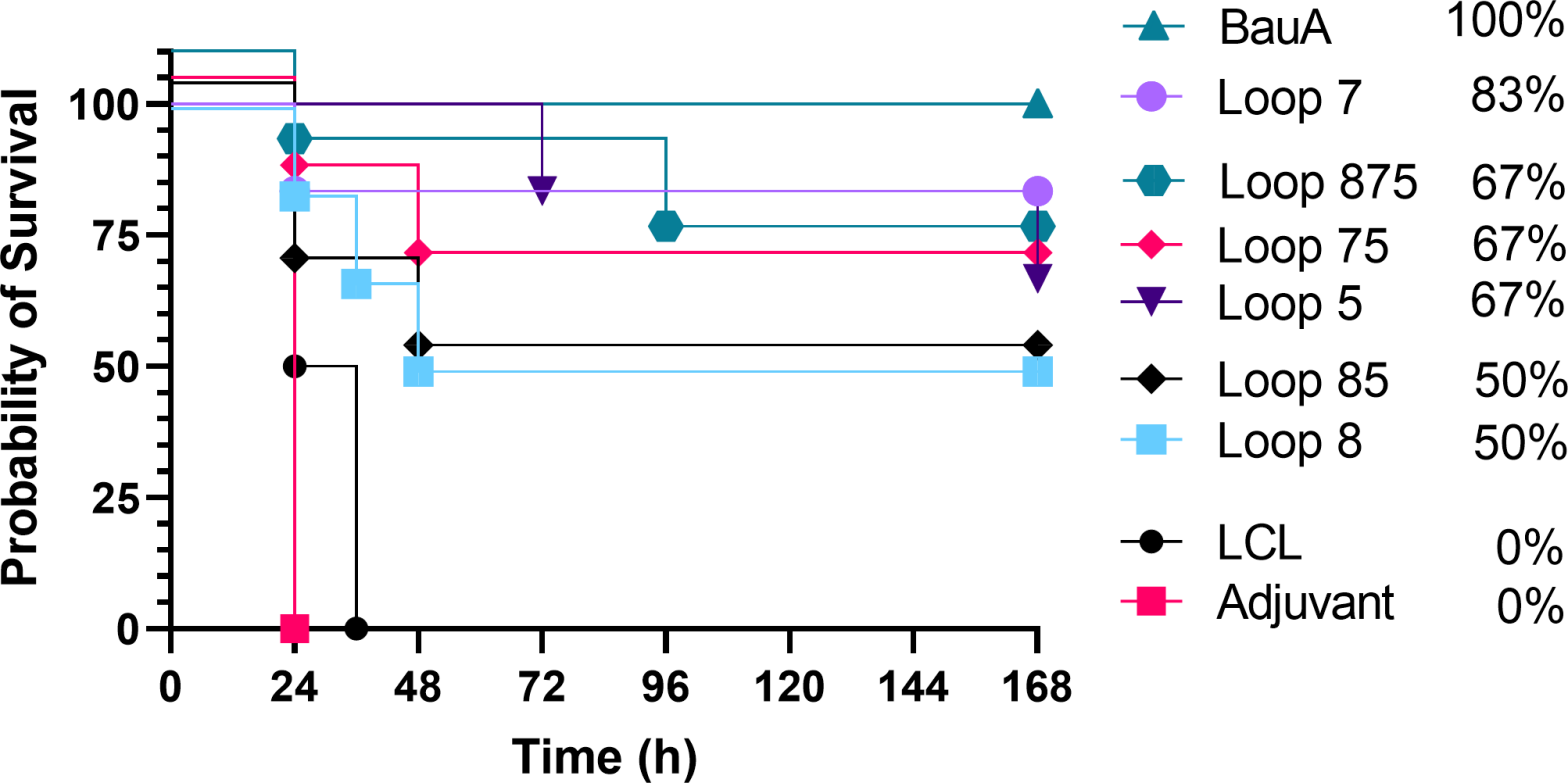
Survival curves of mice passively immunized with serum from mice immunized with different antigens and challenged with a lethal dose of *A. baumannii* ATCC 19606. Mice were challenged with 1.8×10^6^ CFU and monitored for 168 hours (7 days) post challenge for symptoms. Survival curves were analyzed using a non-parametric log rank test and were significant (p<0.0001).

Three weeks after the last immunization (day 49) mice were challenged with a lethal dose of *A. baumannii.* Mice from adjuvant and LCLv3 alone treated groups did not survive or were euthanized within 24 hrs (**Figure 3**). Mice immunized with the hybrid antigen displaying loop 7 and recombinant BauA had a 100% survival rate until the end of the evaluation period. In contrast, mice immunized with hybrid antigens displaying the individual loops 5 and 8 had 66.67% and 50% survival rates, respectively. Perhaps unexpectedly, mice immunized with hybrid antigens displaying loop 7 and an additional one (loops 75, 83.33% survival) or two loops (loops 875, 83.33% survival, did not achieve the level of protection with loop 7 alone. Similarly, the survival rate for the mice immunized with the hybrid antigen displaying loop 5 (66.67%) was not enhanced by the addition of loop 8 (loops 85, 66.67%). To determine whether the protective effect observed in the immunization and challenge study was due to the presence of elicited serum antibodies, a passive immunization of naïve mice and subsequent bacterial challenge experiment was performed (**Figure 4**).

Sera from mice immunized with adjuvant alone or adjuvant with LClv3 were not able to elicit any protection against the bacterial challenge and the same trends were observed in the passive immunization experiment (**Figure 4**) as in the active immunization experiment (**Figure 3**). Passive immunization of naïve mice with anti-BauA mouse serum protected 100% of the mice upon bacterial challenge. Passive immunization of naïve mice with anti-loop 7 hybrid antigen mouse serum protected 83.33% of the mice upon bacterial challenge. The level of survival with antisera against loop 7 was reduced in sera from mice immunized with hybrid antigens with additional loops; loops 75 (66.67%) and loops 875 (66.67%). There was a reduction in the level of survival against loop 5 (66.67%) when a hybrid antigen with an addition loop was present; loops 85 (50%). Taken together the results of both experiments indicate that the protection from infection was primarily mediated by antibodies directed against the individual loops, and that additional loops on the hybrid antigen scaffold does not necessarily result in enhanced protection from infection.

### The impact on bacterial load in the lungs, spleen, and liver

**Figure 5:**
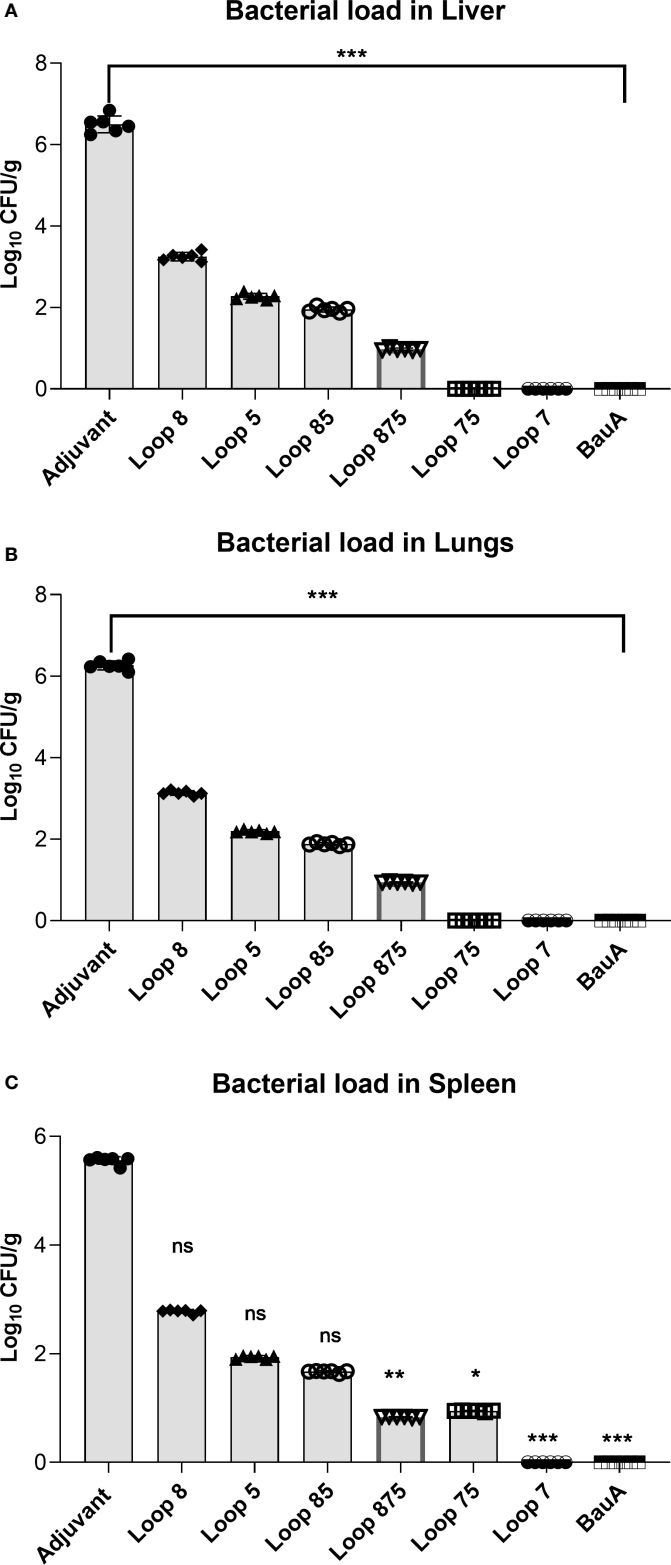
Evaluation of bacterial load in liver **(A)**, lung **(B)**, and spleen **(C)** of immunized mice groups. Mice were challenged with 1.8×10^6^ bacteria and euthanized for tissue collection after 24 hours. Significance is displayed as *p<0.05, **p ≤ 0.01, and ***p<0.001 compared to the adjuvant group. Liver and lungs: Statistically significant differences were determined by One-way ANOVA test followed by Holm-Sidak’s multiple comparison. Spleen showed statistically significant differences as determined by a Kruskal–Wallis test followed by Dunn’s multiple comparison. ns, not significant.

To determine the effect of the elicited immune response on bacterial dissemination, the presence of bacteria in the lungs, spleen and liver 24 hours after bacterial challenge was monitored by colony count (**Figure 5**) in each group of 6 mice. Relatively high CFUs of bacteria were observed in the lungs, spleen, and liver from the adjuvant control treated mice in contrast to mice immunized with the hybrid antigens and recombinant BauA. The observation that immunization with the hybrid antigen displaying BauA loop 7 and the recombinant BauA protein resulted in the virtual absence of colonies in all three tissues examined strongly suggests that the observed colony count in the tissues in the other groups are solely due to *A. baumannii.*

Of the mice immunized with hybrid antigens, the ones displaying loop 7, loops 75 and loops 875 had the lowest burden of *A. baumannii* in all three tissues, suggesting that the immune response against loop 7 was particularly important for clearance in these tissues. Hybrid antigens displaying loop 7 have consistently elicited the strongest protection in both the active and passive immunization studies as well as the bacterial clearance experiment. The efficacy of the anti-Loop 7 mouse serum to protect naïve mice during a lethal bacterial challenge by *A. baumannii* suggest that the clearance in the targeted tissues may have been predominantly by antibody-mediated mechanisms.

## Discussion

The nosocomial pathogen *A. baumannii* is becoming an increasingly serious global health threat. A growing number of community and nosocomial infections including pneumonia, meningitis, bacteremia, sepsis, skin and soft tissue infections, and urinary tract infections demonstrate some of the multiple pathologies and complications caused by this highly successful pathogen (1). The ability of this opportunistic pathogen to resist desiccation and continue to persist in harsh environmental conditions along with the emergence of multi- and pan-resistance to available antibiotic treatments have prompted global calls of concern. There is an urgent need to develop new treatment strategies against *A. baumannii* (5).

An alternative to antibiotic agents that directly act on the bacterium are strategies for enhancing the host’s ability to mount an effective immune response against *A. baumannii* or work in concert with the host’s effector mechanisms, namely vaccines and immunotherapeutic approaches. Whole cells (31) or outer membrane vesicles (32) have been considered for vaccine development but present challenges for achieving comprehensive cross-protection against all potential strains and limit the options that can be considered for immunotherapeutic approaches.

Surface protein antigens are considered attractive vaccine targets as they are often necessary for bacterial survival and virulence thus are exposed to the host immune system. This includes surface antigens that are important for nutrient uptake like zinc and iron, which are in limited quantities in mammalian host environments. While *A. baumannii* can express numerous siderophores to scavenge iron from their environment, expression of acinetobactin (preacinetobactin and acinetobactin) has been shown to be essential for growth of *A. baumannii* on physiological iron sources such as serum, transferrin and lactoferrin (14). Previous studies with mutants in the expression of a gene involved in acinetobactin production or production of BauA in *A. baumannii* strain ATCC 16909 showed defects in interactions with human epithelial cells, killing of insect larvae and survival in a mouse sepsis model (33). More recent studies with strain ATCC 17978 tested a series of deletion mutants in genes involved in the biosynthesis and transport of acinetobactin in a mouse sepsis model demonstrating that BauA was the only essential transport gene whereas deletion of a number of different biosynthesis genes reduced the severity of sepsis (16). Furthermore, immunization of mice with BauA led to the robust production of antibodies and passive immunization with anti-BauA serum protected mice against infection (18). After using structural models to identify the cork and the β-barrel domains, recombinant forms of these proteins were used to generate sera that was reported to provide protection in a passive infection model (34).

Although antigens like the TonB-dependent receptor BauA protein may represent an ideal vaccine target, there are numerous obstacles for the commercial production of integral outer membrane proteins. The yields from production in the outer membrane are relatively low, require detergents or solubilizing and maintaining its stability in aqueous solutions. Although higher levels of production can be attained by production of insoluble inclusion bodies in the cytoplasm, the refolding process and provision of detergents for maintaining solubility present challenges for commercial production. These considerations were what initially led us to develop and use the hybrid antigen approach where we display extracellular loops of membrane proteins constrained by antiparallel beta strands on a foreign, soluble scaffold with the same antiparallel beta strand architecture (19, 20, 24). In the initial step, extracellular loop regions of the C-lobe of the *N. meningitidis* TbpB were removed and replaced to create a LCL that served as a scaffold for displaying epitopes.

The previous results demonstrating that BauA and its subdomains could induce protective antibodies (18, 34) and the B-cell epitope analysis, which identified L4-L9 of BauA as immunogenic (25), was used to guide loop selection. Loops 8, 7, and 5 of BauA (**Figure 1**) appeared well exposed in our structural model thus the DNA encoding these loop regions were inserted into the LCLv3 scaffold at three insertion sites on LCLv3 (**Figure 2**). The LCLv3 scaffold did not induce a protective immune response so that any observed protection from infection could be attributed to the displayed loops.

In the present study all the hybrid antigens induced an immune response that yielded either partial or full protection in mice from sepsis by *A. baumannii*. Notably, the hybrid antigen expressing a single loop (loop 7) was able to protect all mice upon bacterial challenge during the active immunization study and 83% of the mice during the passive immunization study as well as lead to bacterial clearance in the lungs, liver, and spleen, similar to what was observed with immunizations with recombinant BauA (**Figures 3**–**5**). Clearly this indicates that the displayed loop 7 can induce production of antibodies that bind to the conformation of loop 7 present in BauA in a physiologically relevant orientation. The display of additional loops on the hybrid antigen Loop 57 and Loop 857 reduced the protection from sepsis (**Figures 3**–**5**) indicating that the proportion of protective antibodies that are generated is reduced and that any synergistic effect of binding antibodies at different sites is not significant. In other words, the proportion of antibodies directed against the appropriate conformation of loop 7 is reduced when additional loops are available with many conformations capable of binding to B-cell receptors and antibodies. Since the hybrid antigens displaying loop 5 or loops 5 and 8 had a similar level of protection (**Figures 3**–**5**), there clearly is no synergistic effect in this situation either. This contrasts with results in a recent study using the hybrid antigen approach to generate novel antigens targeting the TBDT originally identified as ‘ZnuD’, where single loop hybrids only provided partial protection and a hybrid antigen expressing 4 different loops provided 100% protection (20). At this juncture there does not seem to be any features such as the size of loop that can guide the loop selection process and since there has not been a systematic testing of displayed loops, it is unclear whether bioinformatic approaches can select the most effective loops.

**Figure 6:**
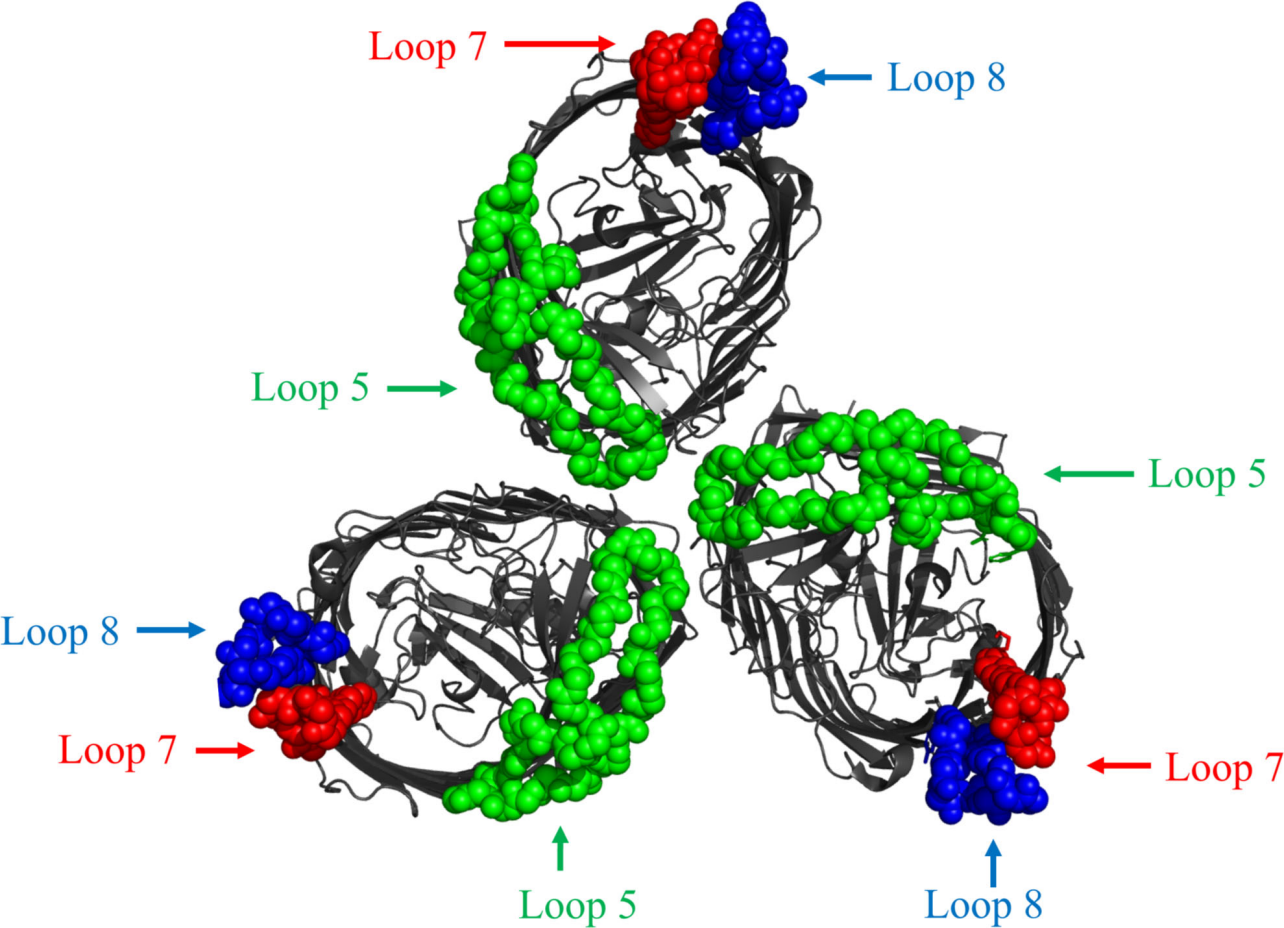
Top-down view of cartoon diagram of structure of BauA trimer complex structure from *A. baumannii* (PDB 6HCP). The beta-barrel is coloured dark gray. The regions of the three loops selected for display on the LCLv3 scaffold are coloured as spheres representing the three selected loops. Loop 5 - green, Loop 7 - red, and Loop 8 – blue.

Recently, the structure of BauA was solved (17) and one of the structures shows BauA as a trimer where the loop 5 extends into the center of the complex and loops 8 and 7 are on the outer edges of the complex (**Figure 6**). Although the trimer could simply be an artefact of the crystallization process, it is important to consider that TonB-dependent processes must occur in gaps in the peptidoglycan layer that would foster some clustering of TBDTs and TonB-ExbB-ExbD complexes (35). In addition, clustering of the proteins involved in TonB-dependent transport could also enhance the efficiency of the process. If the trimer does reflect a natural association in the outer membrane, there would be several antibody-binding sites for either loop 7 or loop 8 in the complex which could result in more effective clearance of the bacteria by the immune system. Although this may provide an explanation as to why loop 7 is efficacious, it does not explain why loop 8 does not elicit similar responses nor why a combination with loop 5 is not synergistic. Clearly the orientation of antibody binding to the native epitope could have a substantial impact on the synergism suggesting that further structural studies are warranted.

Although the results indicate that the hybrid antigen approach is an important tool for targeting critical integral membrane proteins as vaccine antigens, at present there is no systematic approach for selecting loops and loop combinations that can be predicted to be protective. Identifying efficacious hybrid antigens is dependent on evaluating the antigens in animal models. Future continual use of this approach should elucidate the mechanisms to determine a set of rules for developing the best hybrid antigen for every target. Further experiments are required to explore the parameters influencing the optimized induction of a protective immune response.

Granted we were able to provide complete protection from infection using the loop 7 hybrid in this study, the utility of a vaccine against *A. baumannii* faces many challenges. *A. baumannii* infections are prevalent in select populations such as patients in nosocomial settings suffering from skin or burn wounds or military units with combative roles where there is potential for trauma. Although it is possible to vaccinate populations like military units, it may be difficult to initiate an immunization regimen early enough in patients in nosocomial settings prior to their increased risk of infection. In such cases, immunotherapy might be a more plausible option but would involve considerably more research prior to implementation.

In clinical settings, asymptomatic patient colonization is an important reservoir for *A. baumannii* infection and a major risk factor for future symptomatic infections (36–38). *A. baumannii* is commonly isolated from patient skin as well as the upper respiratory tract (36, 39, 40) where access to nutrients like iron is limited (11). *A. baumannii* inhibits the growth of common microbiome skin inhabitants like *Corynebacterium striatum*, *Staphylococcus epidermidis*, *S. hominis* and *S. haemolyticus.* The causative agent of this growth inhibition phenotype is the production of acinetobactin by *A. baumannii* (37) suggesting that acinetobactin may provide a competitive advantage for *A. baumannii* over some commensal bacteria and possibly aid its ability to colonize patients. This phenotype was only seen with acinetobactin production despite the *A. baumannii* strain used encoding other siderophores.

Collectively, this suggests that acinetobactin is not only important in pathogenesis in a murine model, but it may be necessary for colonizing host environments, highlighting the potential importance of BauA, its cognate receptor, for colonization. A target that can elicit a response against BauA may be able to prevent asymptomatic colonization, a notion worth further exploration.

Recently, a vaccine targeting the bacterial receptor for porcine transferrin, a host iron binding protein, expressed by *Glaesserella parasuis*, a porcine pathogen, elicited an immune response that prevented natural colonization in pigs by the targeted bacterial pathogen (41). Therefore, it is tempting to speculate that a rationally designed therapeutic against BauA may be a valuable tool in combatting *A. baumannii* and our results in the present study put forth the hybrid antigen method as a viable means of targeting BauA.

## Data availability statement

The original contributions presented in the study are included in the article/**Supplementary Material**. Further inquiries can be directed to the corresponding author.

## Ethics statement

The animal study was reviewed and approved by Animal Care and Ethical Committee of Shahed University.

## Author contributions

SC was involved in the design of the hybrid antigens, preparation of figures and writing a final draft of the manuscript. RO and MP carried out the animal and immunological analysis experiments. AJ conducted the bioinformatics analysis. VA contributed to SOE PCR experiments. AS and IR conceptualized the study. All authors contributed to the preparation of the manuscript but AS and SC prepared the final manuscript for submission. All authors contributed to the article and approved the submitted version.

## Funding

This work was supported by grant # 1303747 from Shahed University for Iraj Rasooli and Canadian Institutes of Health Research grant number 2016PJT-365835 for Anthony Schryvers.

## Conflict of interest 

AS is an inventor on a patent that describes the design and production of hybrid antigens and is a co-owner of Engineered Antigens Inc.

The remaining authors declare that the research was conducted in the absence of any commercial or financial relationships that could be construed as a potential conflict of interest.

## Publisher’s note

All claims expressed in this article are solely those of the authors and do not necessarily represent those of their affiliated organizations, or those of the publisher, the editors and the reviewers. Any product that may be evaluated in this article, or claim that may be made by its manufacturer, is not guaranteed or endorsed by the publisher.
