# Supplementary material for: Hybrid antigens expressing surface loops of BauA from Acinetobacter baumannii are capable of inducing protection against infection
Source: Front Immunol. 2022 Aug 15;13:933445. doi: 10.3389/fimmu.2022.933445 (PMC9420935; doi:10.3389/fimmu.2022.933445)
Supplement: Supplementary file 1 [file DataSheet_1.zip › Supplementary info/FII Supplementary Figure Captions New.docx]

**Supplementary Figure Captions:**

**Figure S1.** SDS-PAGE analysis of the two soluble single loop hybrid antigens. Lane 1: Protein weight marker Lane 2: Loop 8, Lane 3: Loop 7. Note that all antigens contain a N-terminal HisTEV fusion partner thus are 40 kDa larger than the isolated protein antigen.

**Figure S2.** SDS-PAGE analysis comparing the soluble (s) and insoluble (i) fractions for each single loop hybrid antigen protein. Lane 1. Loop 8 – soluble, Lane 2. Loop 7- soluble, Lane 3. Loop 5-soluble, Lane 4. Loop 8 – insoluble, Lane 5. Loop 7 – insoluble, Lane 6. Loop 7 – insoluble. Note that all antigens contain a N-terminal HisMbpTEV fusion partner thus are 40 kDa larger than the isolated protein antigen.

**Figure S3.** SDS-PAGE analysis comparing the soluble (s) and insoluble (i) fractions for each multi-loop hybrid antigen protein. Lane 1. Loop 75 – soluble, Lane 2. Loop 75- insoluble, Lane 3. Loop 85-soluble, Lane 4. Loop 85 – insoluble, Lane 5. Loop 87 – soluble, Lane 6. Loop 87 – insoluble, Lane 7. Ladder, Lane 8. Loop 875-soluble, Lane 9. Loop 875-insoluble. Note that all antigens contain a N-terminal HisMbpTEV fusion partner thus are 40 kDa larger than the isolated protein antigen.

**Figure S4**. Sera collected from the immunized mice were analyzed to determine the mean endpoint IgG titres against the immunizing protein at each timepoint (D7, D21, D35). Sera from mice from each immunization group (n=6) were assayed in duplicates. Comparisons of mean immunoglobulin titres between different timepoints against each immunizing antigen were performed using two-way ANOVA followed by Tukey’s multiple comparisons test. Serological results are reported as means ± SD, and *p*-values < 0.05 were considered significant (*p<0.05, ** p≤0.01, ***p<0.001, and ****p<0.0001)
