# Supplementary material for: Hybrid antigens expressing surface loops of BauA from Acinetobacter baumannii are capable of inducing protection against infection
Source: Front Immunol. 2022 Aug 15;13:933445. doi: 10.3389/fimmu.2022.933445 (PMC9420935; doi:10.3389/fimmu.2022.933445)
Supplement: Supplementary file 1 [file DataSheet_1.zip › Supplementary info/FII Supplementary Table 1 New.docx]

**Supplementary Table 1. Insoluble vs. Soluble antigens**

| **Soluble form** | **Inclusion body form** | **Protein** |
| --- | --- | --- |
| × |  | LCL |
| × |  | L8 |
| × |  | L7 |
|  | × | L5 |
|  | × | L75 |
|  | × | L85 |
| × |  | L87 |
|  | × | L875 |
