# Supplementary figures and images for: Hybrid antigens expressing surface loops of BauA from Acinetobacter baumannii are capable of inducing protection against infection
Source: Front Immunol. 2022 Aug 15;13:933445. doi: 10.3389/fimmu.2022.933445 (PMC9420935; doi:10.3389/fimmu.2022.933445)

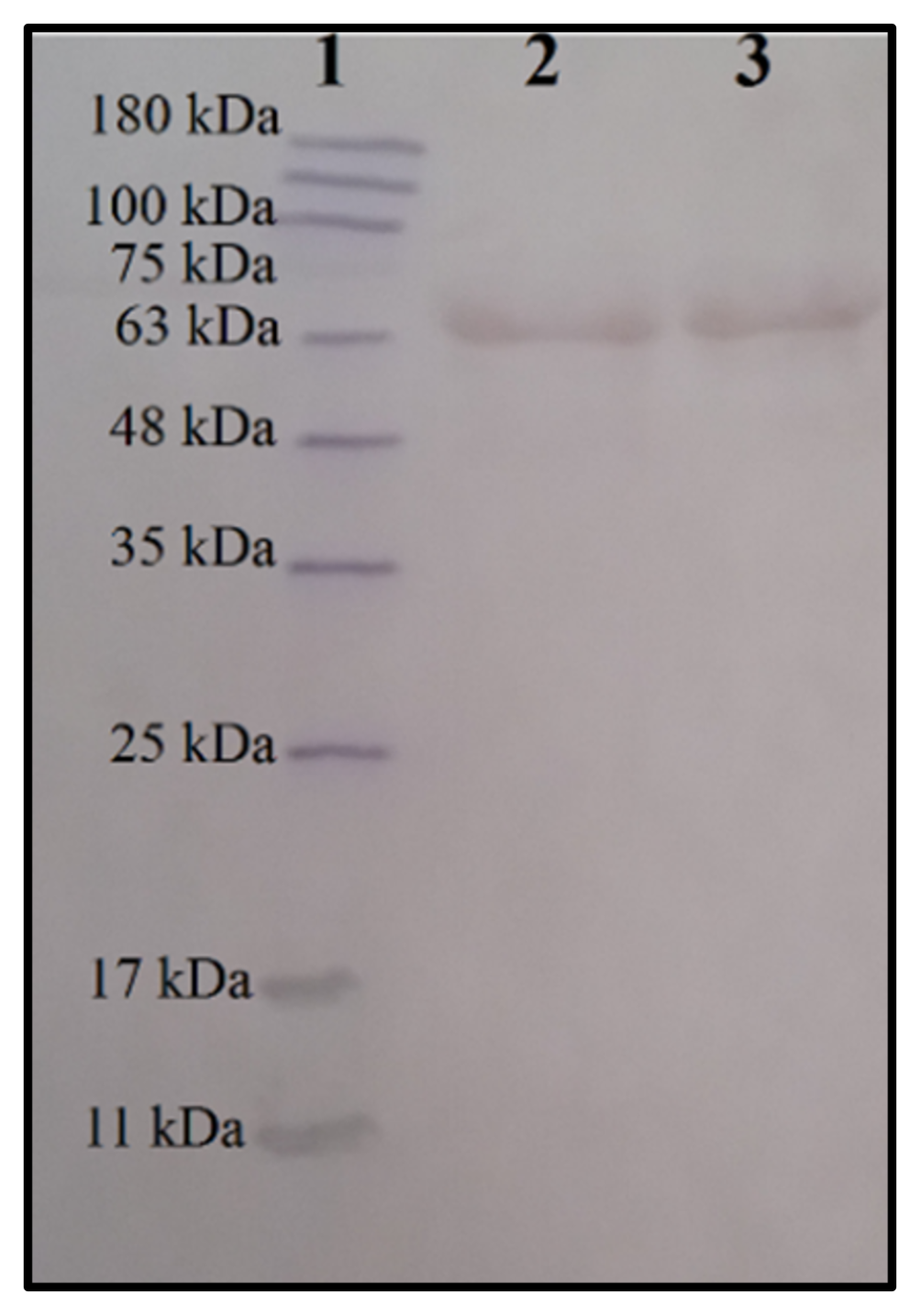

Supplement: Supplementary file 1 [file DataSheet_1.zip › Supplementary info/FII Figure S1 New.tif]

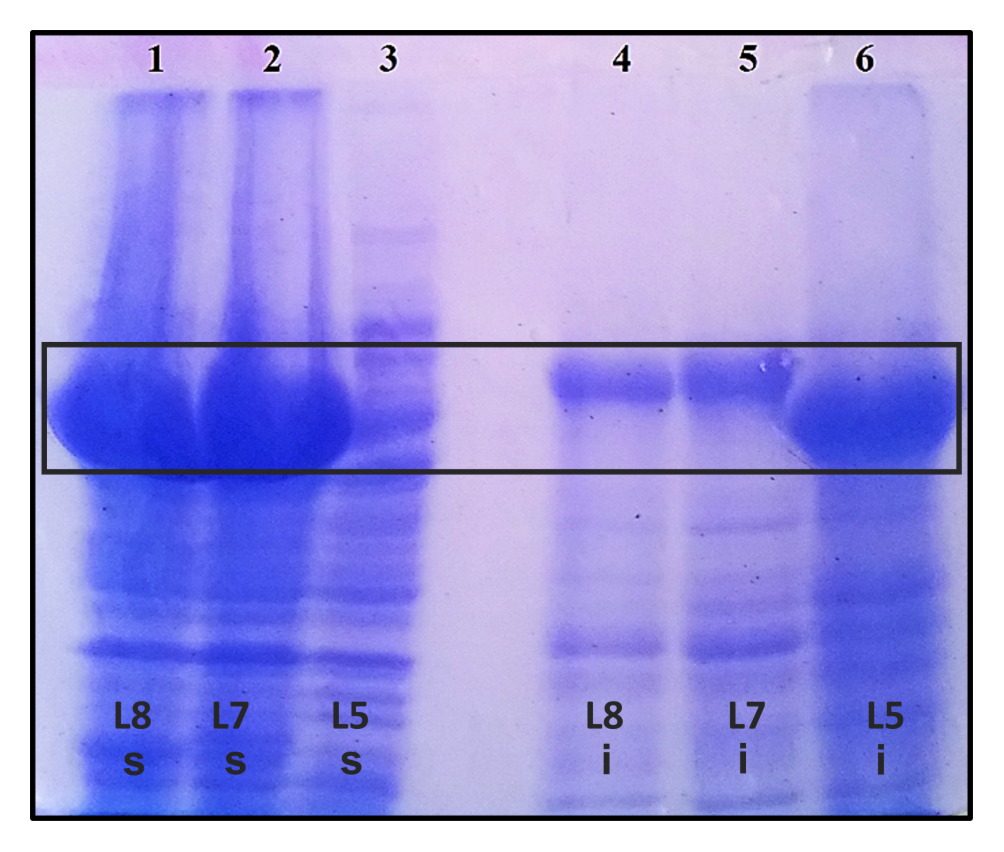

Supplement: Supplementary file 1 [file DataSheet_1.zip › Supplementary info/FII Figure S2 New.tif]

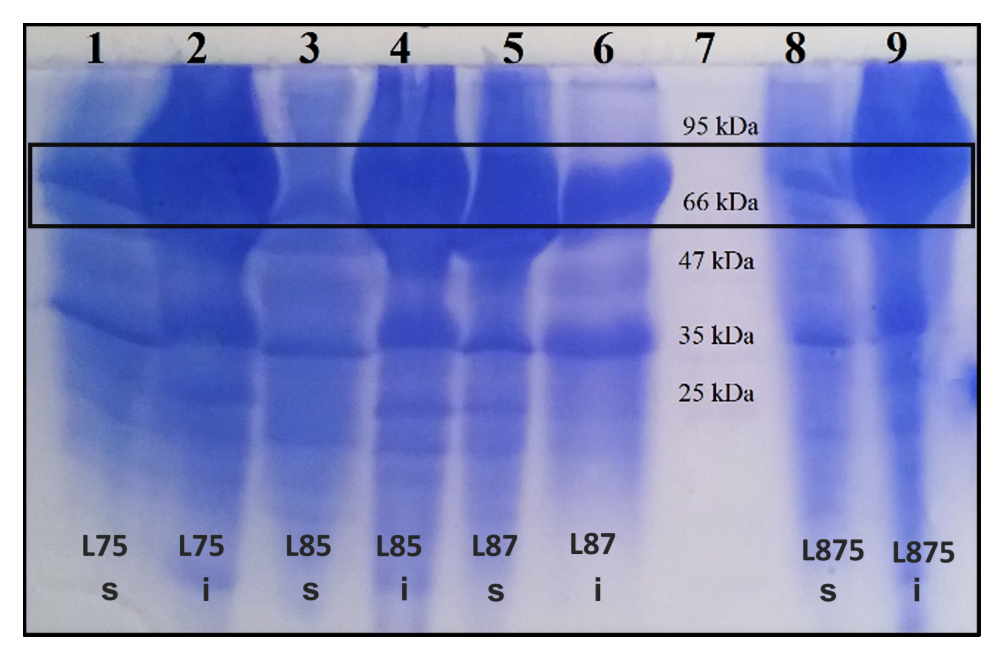

Supplement: Supplementary file 1 [file DataSheet_1.zip › Supplementary info/FII Figure S3 New.tif]

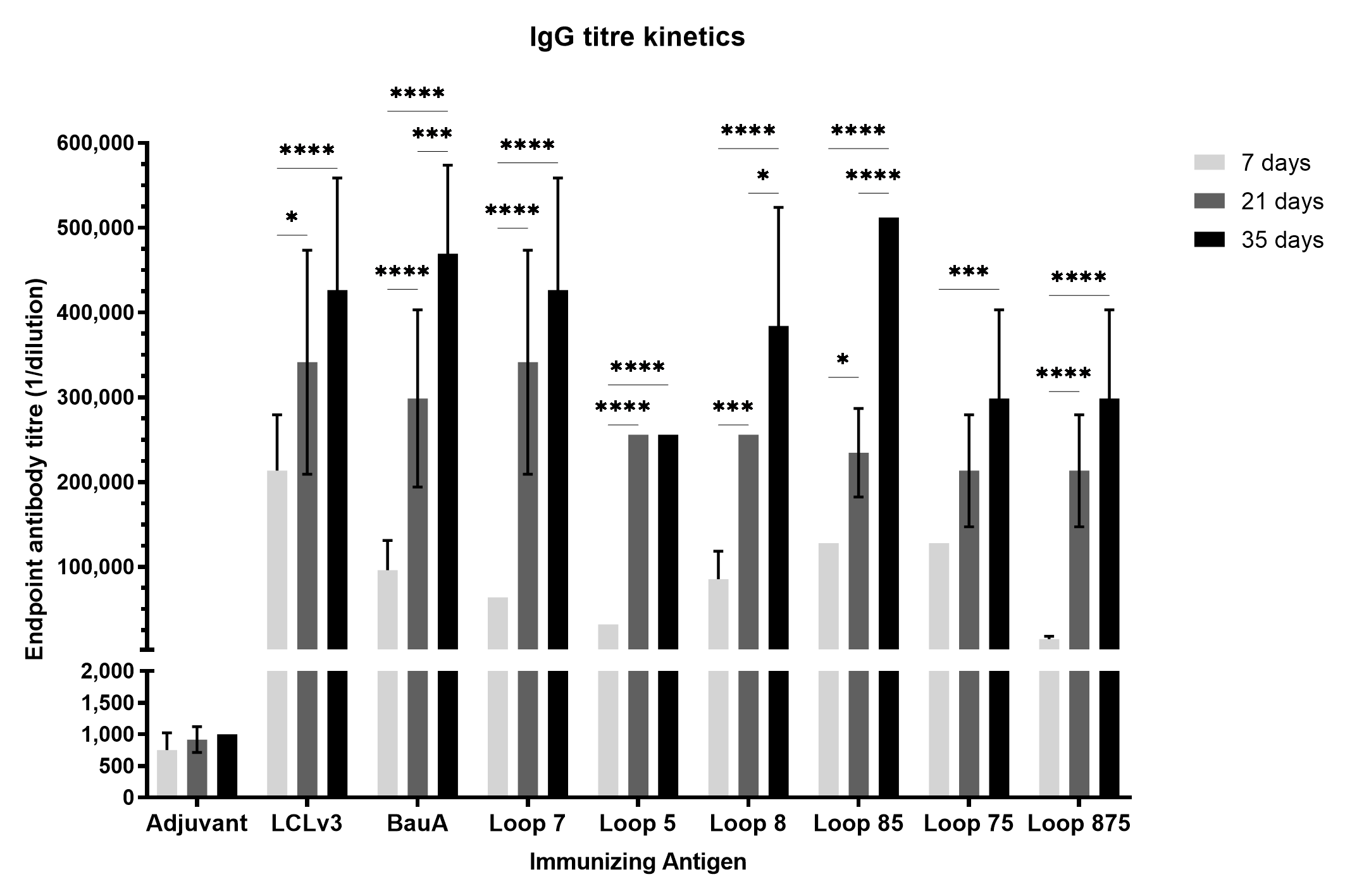

Supplement: Supplementary file 1 [file DataSheet_1.zip › Supplementary info/FII Figure S4 New.tif]
